# *In vitro* irradiation of basement membrane enhances the invasiveness of breast cancer cells

**DOI:** 10.1038/sj.bjc.6604072

**Published:** 2007-11-06

**Authors:** B Paquette, C Baptiste, H Therriault, G Arguin, B Plouffe, R Lemay

**Affiliations:** 1 Department of nuclear medicine and radiobiology, Faculty of medicine and health sciences, Université de Sherbrooke, 3001 12th Avenue North, Sherbrooke, Québec, Canada; 2 Centre de recherche en radiothérapie, CRC-Étienne LeBel, Centre Hospitalier Universitaire de Sherbrooke, 3001 12th Avenue North, Sherbrooke, Québec, Canada; 3 Department of pharmacology, Faculty of medicine and health sciences, Université de Sherbrooke, 3001 12th Avenue North, Sherbrooke, Québec, Canada J1H 5N4; 4 Ottawa Health Research Institute, Moses and Rose Loeb Research Centre, 725 Parkdale Avenue, Ottawa, Ontario, Canada K1Y 4E9

**Keywords:** breast cancer, basement membrane, invasion, matrix metalloproteinase, matrikine, radiation

## Abstract

Following removal of the primary breast tumour by conservative surgery, patients may still have additional malignant foci scattered throughout the breast. Radiation treatments are not designed to eliminate all these residual cancer cells. Rather, the radiation dose is calculated to optimise long-term results with minimal complications. In a tumour, cancer cells are surrounded by a basement membrane, which plays an important role in the regulation of gene expression. Using an invasion chamber, we have shown that irradiation before cell plating of a reconstituted basement membrane (Matrigel; Becton Dickinson, Bedford, MA, USA) increased the invasiveness of the breast cancer cells MDA-MB-231. This radiation enhancement of invasion was associated with the upregulation of the pro-invasive gene matrix metalloproteinase (MMP)-2. The expression of membrane type 1 matrix metalloproteinase (MT1-MMP) and tissue inhibitor of metalloproteinase-2 (TIMP), which are required to activate the MMP-2, were also increased. Confirming the role of MMP-2 and MT1-MMP, radiation enhancement of cancer cell invasion was prevented by an MMP-2 inhibitor and an anti-MT1-MMP antibody. This study also demonstrated that radiation can potentially enhance the invasion ability by inducing the release of pro-invasive factors stored in the Matrigel. Conversely, no enhancement of invasiveness was observed with the low metastatic cell line MCF-7. This lack of invasiveness correlated with the absence of the MMP-2 activator MT1-MMP in the MCF-7 cells. Radiotherapy is an efficient modality to treat breast cancer which could be further improved by inhibiting the pro-invasive gene upregulated by radiation.

The efficiency of radiotherapy to treat breast cancer is well established (Clarke *et al*, 2005). However, the radiation dose used in the clinic (50–60 Gy) is not calculated to eliminate all cancer cells scattered throughout the breast but rather to optimise long-term results with minimal complications. The three major normal tissue side-effects are cosmetic outcome, cardiac complications and pulmonary fibrosis (Coles *et al*, 2005). The principal long-term complications that impair cosmetics are fibrosis and induration of the breast resulting from inflammatory responses induced by radiation (Archambeau *et al*, 1995). The probability of observing late changes by 5 years for patients receiving radiotherapy is about 40% (Coles *et al*, 2005).

Following surgery both surgeons and patients are reassured by histological confirmation of complete excision, with clear margins. However, several reports have questioned the accuracy of pathological assessment (Holland *et al*, 1985; Frazier *et al*, 1989; Silverstein *et al*, 1994). In a detailed analysis of mastectomy specimens, it has been shown that malignant foci were found at a distance of 2 cm from tumour edge in 42% of specimens (Holland *et al*, 1985). An other team has reported that residual cancer cells were observed in as many as 26% of patients reported to have pathological margins clear of tumour (Frazier *et al*, 1989). Of patients clinically and mammographically suspected of having unifocal breast cancer, 39–63% will have additional malignant foci in the ipsilateral breast as determined by detailed serial sectioning of the mastectomy specimen (Lagios *et al*, 1981; Holland *et al*, 1985).

Consequently, a number of breast cancer cells are not eliminated after surgery and radiotherapy. These residual cancer cells could be responsible for distant metastases associated with death from breast cancer. The overall aim of this study is to determine whether ionising radiation could enhance the invasion capacity of the breast cancer cells that are not eliminated after therapy.

A breast tumour can be divided into five parts: cancer cells, fibroblast, inflammatory cells, blood vessels and the basement membrane. In this study, we want to determine whether irradiation of basement membrane could enhance the invasion ability of breast cancer cells. Basement membrane acts as a barrier to cancer cell invasion. To overcome this barrier, advancing cells express proteinases and/or proteinase activators at their leading edge, where complex proteolysis can direct cell migration. Among these proteinases, matrix metalloproteinases (MMPs) are believed to play a major role in tumour invasion since they can degrade almost all the basement membrane macromolecules (Rooprai and McCormick 1997; Fillmore *et al*, 2001).

Matrix metalloproteinase-2 plays a major role in the proteolysis of basement membrane and is associated with tumour invasion and metastasis (Gilles *et al*, 1998; Jones *et al*, 1999). Matrix metalloproteinase-2 is released by cancer cells or fibroblasts under an inactive form called proMMP-2 (Liotta and Stetler-Stevenson 1991; Belkhiri *et al*, 1997). Activation of proMMP-2 requires the contribution of a cell membrane MMP (ie membrane type 1 MMP, MT1-MMP) and the tissue inhibitor of metalloproteinase-2 (TIMP-2). The catalytic domain of MT1-MMP binds to the N-terminal portion of TIMP-2, leaving the negatively charged C-terminal region of TIMP-2 available for binding to the hemopexin-like domain of proMMP-2 (Zucker *et al*, 1998). This ternary complex clusters proMMP-2 near a TIMP-2-free MT1-MMP molecule on the cell surface. Free MT1-MMP cleaves the prodomain of proMMP-2 thus generating the 64-kDa activation intermediate that then binds to *α*_v_*β*_3_ integrin and matures into the fully active 62-kDa MMP-2. Localisation of active MMP-2 and *α*_v_*β*_3_ integrin at the migration front accelerates cancer cell migration (Brooks *et al*, 1996; Deryugina *et al*, 2001).

Basement membrane stores pro-invasive factors such as insulin-like growth factor (IGF), epidermal growth factor (EGF) and basic fibroblast growth factor (bFGF) which can stimulate the expression of MMP-2 and other MMPs (Mira *et al*, 1999; Zhang and Brodt 2003; Lee *et al*, 2004). Injuries to the basement membrane can result in the release of these pro-invasive compounds. Furthermore, fragmentation of matrix proteins by free radicals or limited proteolysis can generate biologically active peptides, that is, the matrikines (Vartio 1989; Monboisse and Borel 1992; Raats *et al*, 1997; Duca *et al*, 2004). Once released, these matrikines, such as elastin peptides, can increase the expression of MMP-2, MT1-MMP and TIMP-2 in fibrosarcoma HT-1080 cells (Brassart *et al*, 1998). Matrikines can also be derived from laminins, proteoglycans or collagens (Pasco *et al*, 2004).

In this study, we have determined whether irradiation of a reconstituted basement membrane (Matrigel; Becton Dickinson, Bedford, MA, USA) could enhance the expression of MMP-2, MT1-MMP and TIMP-2 from breast cancer cells. Invasion chambers coated with Matrigel were irradiated prior to plate breast cancer cells and then the invasiveness of a highly metastatic breast carcinoma cell line, the MDA-MB-231 cells and of a weakly metastatic one, the MCF-7 cells were determined.

## MATERIALS AND METHODS

### Chemicals and reagents

*p-*Aminophenylmercuric acetate (APMA), Cell Dissociation Solution, diethyl pyrocarbonate (DEPC), DNase and gelatine were purchased from Sigma-Aldrich Canada Ltd (Oakville, Ontario, Canada). (2R)-[(4-biphenylylsulfonyl)amino]-*N*-hydroxy-3-phenylpropionamide, fluorogenic peptide MMP Substrate III, proMMP-2 and -9 were obtained from Calbiochem (San Diego, CA, USA). Matrigel (standard growth factors), invasion chambers and Cell Recovery Solution were obtained from BD Biosciences (Oakville, Ontario, Canada).

### Mammary cells culture

The MDA-MB-231 and MCF-7 human breast cancer lines were obtained from American Type Culture Collection. Culture medium consisted of minimum essential medium (MEM) containing phenol red (Sigma, A-6770) and supplemented with 10% fetal bovine serum (FBS), penicillin (10 U ml^−1^), streptomycin (10 *μ*g ml^−1^), amphotericin B (0.25 *μ*g ml^−1^), L-glutamine (2 mM) and sodium pyruvate (1 mM).

### Irradiation of Matrigel

A volume of 500 *μ*l of Matrigel diluted in MEM medium without FBS at a final concentration of 5 mg ml^−1^ was added in six-well plate and allowed to polymerise for 1 h at 37°C. Matrigel covered by 0.5 ml of phosphate-buffered saline (PBS) was irradiated using the ^60^Co source (Gammacell 220, Nordion, Canada) at the dose indicated. Non-irradiated Matrigel was used as control.

### RNA isolation

Breast cancer cells were plated on either irradiated or non-irradiated Matrigel and incubated for 24 h in FBS-free culture medium. Cells were isolated with 1 ml Cell Recovery Solution (Sigma) incubated at room temperature for 10 min. Total RNA was isolated using the RNeasy Mini kit (QIAGEN, Mississauga, Canada) and then quantified in DEPC-treated water with a spectrophotometer. To reduce non-specific amplification, RNA samples were treated with the Amplification Grade DNase I kit (Sigma, Oakville, ON, Canada). Briefly, 1 *μ*g of RNA diluted in 8 *μ*l DEPC water was treated with 1 *μ*l Amplification Grade DNase I (1 U *μ*l^−1^) in a final volume of 10 *μ*l completed with the reaction buffer (20 mM Tris-HCl pH 7.5, 1 mM CaClB_2_). After 15 min incubation at room temperature, 1 *μ*l of the Stop solution (50 mM EDTA) was added. The RNA samples were then heated at 70°C for 10 min to denature the DNase I.

### Real-time PCR

cDNA was produced from the DNase I-treated RNA samples by adding to 1 *μ*g RNA, the reverse transcriptase M-MLV (200 U per 21 *μ*l) (Bio Can Scientific Inc., Mississauga, ON, Canada), the RNase inhibitor RNAguard™ (2 U per 21 *μ*l) (Amersham Biosciences, Baie d'Urfe, Québec, Canada), the pd(N)6 (100 pmol *μ*l^−1^) and the dNTPS (10 mM) (Amersham Pharmacia Biotech Inc., Piscataway, NJ, USA) in a final volume of 21 *μ*l completed with the reaction buffer (final concentrations: 50 mM Tris-HCl pH 8.3, 75 mM KCl, 3 mM Mgcl_2_, 10 mM dithiothreitol). The reaction conditions were 20°C for 10 min, 42°C for 50 min and 95°C for 5 min.

A total of 50 ng of the cDNA product was used for real-time PCR. Amplification of each PCR product was carried out separately in a different tube. The primers used were MMP-2 (71pb) sense primer 5′-CGCTCAGATCCGTGGTGAG-3′, antisense primer 5′-TTGTCACGTGGCGTCACAG-3′ (Strup-Perrot *et al*, 2004); MT1-MMP (496pb) sense primer, 5′-CGCTACGCCATCCAGGGTCTCAAA-3′, antisense primer 5′-CGGTCATCATCGGGCAGCACAAAA-3′ (Daja *et al*, 2003); TIMP-2 (303pb) sense primer 5′-GGTCTCGCTGGACGTTGGAG-3′, antisense primer 5′-GGAGCCGTCACTTCTCTTG-3′ (Balduyck *et al*, 2000). As control, glyceraldehyde-3-phosphate dehydrogenase (GAPDH) (307pb) amplification was performed using the primers: sense 5′-CGGAGTCAACGGATTTGGTCGTAT-3′, antisense 5′-AGCCTTCTCCATGGTGGTGAAGAC-3′ (Wong *et al*, 2001).

Real-time PCR were performed using the detection kit Quantitect™ SYBR® Green PCR® (QIAGEN) under the following conditions. Reaction mixtures with primers at 10 pmol *μ*l^−1^ were incubated in the thermocycler LightCycler (Roche Applied Science, Laval, Québec, Canada). All PCR programs started with 15 min at 95°C to activate the HotStarTaq® DNA polymerase. The specific conditions were MMP-2, 30 s at 94°C, 45 s at 52°C and 1 min at 72°C; MT1-MMP, 30 s at 94°C, 30 s at 62°C and 1 min at 72°C; TIMP-2 and GAPDH, 50 s at 94°C, 50 s at 55°C and 20 s at 72°C. Amplifications were performed for a maximum of 45 cycles. Relative quantification was performed with the LightCycler® Software 4.0 according to the manufacturer's instructions and the method reported by Pfaffl (2001). Fusion curves were performed to eliminate the possibility of quantifying non-specific sequences. The PCR products were also analysed on 1.8% agarose gels to confirm that the right amplifications had been effectuated. DNA bands were stained with the fluorogen SYBR Green IP^®P P^(Molecular Probes, Burlington, Ontario, Canada) and analysed at 500 nm with the STORM® 860 and the software ImagQuant™ (Molecular Dynamics Inc., Baie d'Urfe, Québec, Canada).

### Secretion of MMP-2 from cancer cells plated on irradiated Matrigel

A volume of 500 *μ*l of Matrigel diluted in an MEM medium without FBS at a final concentration of 5 mg ml^−1^ was added to the six-well plate and allowed to polymerise for 1 h at 37°C. Matrigel covered by 0.5 ml of PBS was irradiated using the ^60^Co source (Gammacell 220, Nordion) at a dose of 20 Gy. Non-irradiated Matrigel was used as control. MDA-MB-231 or MCF-7 breast cancer cells (6 × 10^5^) were added on Matrigel in 10% FBS MEM and incubated for 3 h. Then the cells were washed twice using PBS, and incubated for 24 h in FBS-free culture medium. Conditioned media were obtained using FBS-free culture medium to eliminate the endogenous proMMPs found in FBS. The conditioned media were concentrated 20 times using the Centricon YM30 column (Millipore, Etobicoke, Ontario, Canada) and samples were then analysed on a zymography gel as previously described (Paquette *et al*, 2005). Zymography is a non-reducing acrylamide gel polymerised with an MMP-2 and MMP-9 Substrate, gelatine. It allows proMMP and MMP to be distinguished according to their molecular weights. Samples were applied on a 12% polyacrylamide-sodium dodecyl sulfate (SDS) gel containing 0.1% gelatine and electrophoresed at 150 V during 3 h at 4°C. After removal of SDS from the gel by incubating in 2.5% Triton X-100 (30 min, four times), the gel was incubated at 37°C for 18 h in 40 mM Tris-HCl, pH 7.5, containing 10 mM CaCl_2_, 1 *μ*M ZnCl_2_, 200 mM NaCl and stained with Coomassie blue R-250 (Albini *et al*, 1995). As positive control, MMP-2 and -9 obtained from Calbiochem were activated with 1 mM APMA as previously described (Paquette *et al*, 2003) and analysed by zymography. Briefly, 17 ng of proMMPs were incubated in 10 *μ*l of buffer A (100 mM Tris-HCl pH 7.6, 150 mM NaCl, 5 mM Cacl_2_, 1 *μ*M Zncl_2_, 0.01% Brij 35) containing 1 mM APMA at 37°C for 2 h. A stock solution of 10 mM APMA was prepared in 0.1 N NaOH. MMPs activated by APMA were diluted 1 : 8 and then electrophoresed. Gels were scanned and analysed using ImageJ 1.34n (public domain Java image, NIH, Bethesda, MD, USA).

### Invasion chambers

Invasion chambers coated with Matrigel were rehydrated with 1 ml MEM 0.1%. bovine serum albumin (BSA) for 1 h at 37°C. Before cell plating, the chambers were irradiated at the indicated dose using a ^60^Co source (Gammacell 220, Nordion). MDA-MB-231 and MCF-7 breast cancer cells were harvested with 1 ml of Cell Dissociation Solution (Sigma) incubated for 10 min at room temperature, washed with PBS, suspended in 1 ml MEM 0.1% BSA, plated (1 × 10^4^ or 4 × 10^4^ in 0.5 ml) in the invasion chambers and then incubated at 37°C for 6 h. In another series, breast cancer cells were treated in the invasion chambers with 0.1 mM of the MMP-2 inhibitor (2R)-[(4-biphenylylsulfonyl)amino]-*N*-hydroxy-3-phenylpropionamide. To determine the role of MT1-MMP, breast cancer cells were mixed with an anti-MT1-MMP antibody (Fitzgerald Industries International Inc., catalogue no. RDI-MMP14 HABR) at 20 *μ*g ml^−1^ before plating in irradiated invasion chambers. As chemoattractant, MEM medium supplemented with 10% FBS was added in the lower compartment of the invasion chamber. Cells, which have crossed the Matrigel and the porous membrane, were fixed, stained with 0.5% crystal violet and counted under the microscope.

To determine whether the enhancement of breast cancer cell invasion was caused by invasion factors released from irradiated Matrigel, four conditions were tested: (1) invasion through unirradiated Matrigel; (2) invasion through Matrigel which was irradiated without a layer of PBS before cell plating; (3) invasion through Matrigel which was irradiated with a layer of PBS (0.2 ml) before cell plating and (4) invasion through unirradiated Matrigel where 0.2 ml of conditioned PBS, isolated from an irradiated invasion chamber was added. This latter conditioned PBS covered the Matrigel of an invasion chamber during the irradiation. For the conditions 1 and 2, 0.2 ml PBS was added just before cells plating.

### Irradiation of proMMP-2

Cancer cells release inactive proMMP-2 which can be activated by free radicals, such as produced by the radiolysis of water (Saari *et al*, 1992; Paquette *et al*, 2003). To determine whether radiation could activate proMMP-2, aliquots (0.17 *μ*g) of this protease (Calbiochem) were irradiated using a ^60^Co source (Gammacell 220, Nordion) to a final dose of 20 Gy in the buffer used for the enzymatic assay. Conversion from proMMP-2 to active MMP-2 was monitored according to the cleavage of a fluorogenic peptide. Briefly, irradiated proMMP-2 was mixed in the reaction buffer A (50 mM borate pH 7.4, 5 mM CaCl_2_, 20% glycerol, 0.005% Brij, 0.01 mM ZnCl_2_) to a final volume of 20 *μ*l. The samples were then incubated with 50 *μ*M of the fluorogenic peptide MMP Substrate III (Calbiochem). The enzymatic activity was recorded according to the variation of RFU per sec. Care was taken to always work at the saturated concentration of the fluorogenic peptide. The kinetics of peptide cleavage were then followed for 30 min using the 96-well plate reader Synergy HT (BioTek Instruments, Winooski, VT, USA) set at a λex=340 nm and a *λ*em=485 nm. As a positive control, proMMP-2 was activated with 1 mM APMA as previously described (Paquette *et al*, 2003).

### MMP-2 activity from breast cancer cells surface

MMP-2 activity on MDA-MB-231 and MCF-7 cells plated on irradiated Matrigel was measured using the fluorogenic peptide MMP Substrate III. Matrigel was irradiated at 20 Gy and then the breast cancer cells were plated and incubated for 18 h at 37°C. The MEM 0.1% BSA was replaced by the enzymatic buffer A and the MMP-2 activity was measured by adding the fluorogenic peptide as previously described in the section ‘Irradiation of proMMP-2’.

### Statistical analysis

Data are expressed as the mean±standard deviation and analysed using a Student's *t*-test by comparing the experimental value to the non-irradiated control.

## RESULTS

### Irradiated Matrigel enhances the expression of MMP-2, MT1-MMP and TIMP-2

The coordinate expression of MMP-2, MT1-MMP and TIMP-2 is required for the activation of proMMP-2. A zymography gel was performed to determine whether irradiation of Matrigel (reconstituted basement membrane) can increase the secretion of MMP-2 from breast cancer cells. Petri dishes coated with Matrigel were irradiated at 20 Gy using a ^60^Co irradiator. Two cell lines were tested: the MDA-MB-231 cells which are highly metastatic and the weakly metastatic MCF-7 cells. They were plated on Petri dishes after irradiation of Matrigel. The conditioned media were harvested 24 h later and MMP-2 was quantified by zymography gel. As a control, breast cancer cells were plated on non-irradiated Matrigel.

**Figure 1:**
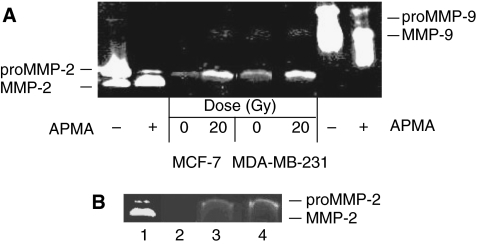
Irradiated Matrigel increased the secretion of matrix metalloproteinase (MMP)-2 from MDA-MB-231 and MCF-7 breast cancer cells. (**A**) Cancer cells (6 × 10^5^) were added on a layer of Matrigel which was previously irradiated using a ^60^Co source at 20 Gy. Non-irradiated Matrigel was used as control. After 24 h incubation, the culture media was removed, concentrated and analysed by zymogram electrophoresis gel. As controls, proMMP-2 and -9 obtained from Calbiochem were activated with *p*-aminophenylmercuric acetate (APMA) and the activated and unactivated forms were added to the zymogram gel. (**B**) MMP-2 released from Matrigel following irradiation. (1) proMMP-2 activated by APMA; (2) concentrated FBS-free culture media; (3) concentrated FBS-free culture media incubated for 24 h on non-irradiated Matrigel; and (4) concentrated FBS-free culture media incubated for 24 h on Matrigel previously irradiated at 20 Gy. These assays were repeated four times.

As shown in Figure 1A, irradiation of Matrigel at 20 Gy increased by about fourfold the level of proMMP-2 released into the culture media by MDA-MB-231 and MCF-7 breast cancer cells, compared to the non-irradiated controls. Conversely, the quantity of MMP-9 secreted under these conditions was negligible (Figure 1A).

Since proMMP-2 can be stored in Matrigel, the zymography was repeated to determine whether ionising radiation could induce the release of proMMP-2 from Matrigel. Our results showed that very few proMMP-2 was stored in Matrigel and radiation at 20 Gy resulted in only a slight increase of proMMP-2 released (Figure 1B lane 3 *vs* 4). This data also confirmed that proMMP-2 was absent from the FBS-free culture media (Figure 1B, lane 2).

ProMMP-2 is activated on the surface of breast cancer cells by the MT1-MMP and TIMP-2. Their corresponding mRNA expressed by MDA-MB-231 and MCF-7 cells was quantified using a real-time PCR assay (Table 1). The expression of both MT1-MMP and TIMP-2 by MDA-MB-231 cells plated on irradiated Matrigel were significantly increased by 3.07-fold and 1.59-fold, respectively. Conversely, MT1-MMP was not detectable in the weakly metastatic cells MCF-7, while the level of TIMP-2 was not significantly increased by the irradiated Matrigel. Regarding the MMP-2, its expression was stimulated in both cell lines plated on irradiated Matrigel, supporting the results obtained with the zymography analysis.

### Enhancement of MDA-MB-231 cells invasion capacity

Invasion chambers were then used to determine whether irradiation of Matrigel can increase the invasiveness of breast cancer cells. Invasion chambers contain an 8 *μ*m pore-size membrane coated with Matrigel. MMPs can digest the Matrigel, which allows the invasion of cancer cells through the other side of the porous membrane. Prior to the addition of breast cancer cells, invasion chambers were irradiated using a ^60^Co source at the dose indicated.

**Figure 2:**
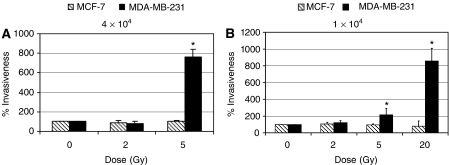
Enhancement of invasiveness induced by ionising radiation. Matrigel coated in the invasion chambers was irradiated using a ^60^Co source. MDA-MB-231 and MCF-7 cells (**A**: 4 × 10^4^ or **B**: 1 × 10^4^) were added on the Matrigel after the irradiation and then incubated for 6 h in MEM 0.1% BSA. MDA-MB-231 cells: ^*^n=4; 0 *vs* 5 Gy: *P*=0.05; 0 *vs* 20 Gy: *P*=0.0004.

Results shown in Figure 2 were normalised according to the number of cells, which have crossed the Matrigel layer in absence of radiation. Our data demonstrated that irradiation of Matrigel at a dose as low as 5 Gy markedly increased the invasiveness of MDA-MB-231 cells (Figure 2A). An enhancement of 7.5-fold was measured when the MDA-MB-231 cells were added at a concentration of 4 × 10^4^ per 0.5 ml. At radiation doses higher than 5 Gy, the number of cells crossing the Matrigel layer was too large to be accurately counted. Conversely, no enhancement of invasion following Matrigel irradiation was observed for the weakly metastatic MCF-7 cells.

To determine the effect of higher radiation dose on the invasiveness of breast cancer cells, the assay was repeated at a lower concentration of cancer cells, that is, 1 × 10^4^ cells per 0.5 ml (Figure 2B). At a radiation dose of 5 Gy, the invasion of MDA-MB-231 cells still increased by about twofold, while an enhancement of 8.5-fold was obtained following an irradiation at 20 Gy. The higher enhancement of radiation-induced invasion at 5 Gy observed after plating 4 × 10^4^ compared to 1 × 10^4^ cells was suggested to be associated with a higher quantity of MMP-2 secreted by the MDA-MB-231 cells. As previously measured, the enhancement of invasiveness was observed only with the MDA-MB-231 cells since irradiation of Matrigel did not modify the invasion capacity of the low metastatic MCF-7 cells.

**Figure 3:**
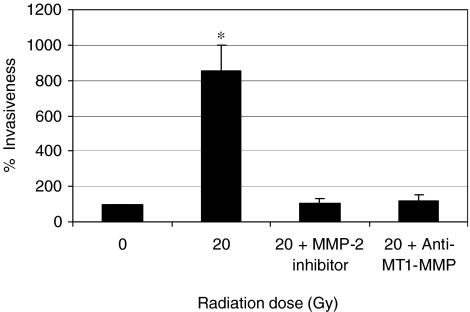
A matrix metalloproteinase (MMP)-2 inhibitor or an anti-membrane type 1 matrix metalloproteinase (MT1-MMP) antibody prevented radiation enhancement of MDA-MB-231 breast cancer cell invasion. MDA-MB-231 cancer cells were mixed with either the MMP-2 inhibitor (2R)-[(4-biphenylylsulfonyl)amino]-*N*-hydroxy-3-phenylpropionamide at 0.1 mM, or an anti-MT1-MMP antibody (20 *μ*g ml^−1^) before plating in the invasion chamber which was previously irradiated at 20 Gy.^*^n=3, *P*<0.05.

The real-time PCR assay has demonstrated that irradiated Matrigel increased the expression of MMP-2 and MT1-MMP. To further investigate the role of theses MMPs in radiation enhancement of invasion, the invasion assay was repeated in presence of either an MMP-2 inhibitor, the (2R)-[(4-biphenylylsulfonyl)amino]-*N*-hydroxy-3-phenylpropionamide or an anti-MT1-MMP antibody (20 *μ*g ml^−1^). As shown in Figure 3, both the MMP-2 inhibitor and anti-MT1-MMP antibody significantly prevented the enhancement of MDA-MB-231 cells invasion induced by ionising radiation.

### Release of pro-invasive factors from irradiated Matrigel

Some modifications to Matrigel can be produced by the direct interaction of radiation with Matrigel. On the other hand, the radiolysis of water generates free radicals, which can also damage the Matrigel. To determine the importance of these pathways, invasion chambers were irradiated with or without a layer of PBS added on the Matrigel. As seen in Table 2, irradiation of invasion chambers without PBS has increased by fourfold the number of MDA-MB-231 cells that have crossed the Matrigel, compared to data obtained with non-irradiated invasion chambers (condition no. 1 *vs* 2). This enhancement of the invasiveness of the breast cancer cells was further increased when the invasion chambers were covered by a layer of PBS during exposure to radiation, that is, an 8.5-fold increase compared to non-irradiated control (condition no. 1 *vs* 3). These data suggest that ionising radiation does induce some modification of the Matrigel, which enhances the invasiveness of MDA-MB-231 cells.

The invasion assay was then repeated to determine whether pro-invasive factors stored in the Matrigel could be released by the ionising radiation. To verify this hypothesis, invasion chambers covered by a layer of PBS were irradiated. These conditioned PBS were then transferred to new invasion chambers, where MDA-MB-231 cells were then added (condition no. 4). As seen in Table 2, conditioned PBS isolated from irradiated Matrigel increased by more than eightfold the number of MDA-MB-231 cells that have crossed the Matrigel compared to non-irradiated invasion chambers (condition no. 1 *vs* 4). These data suggest that ionising radiation can induce the release of pro-invasive factors stored in Matrigel, which can enhance the invasiveness of MDA-MB-231 breast cancer cells.

### Enhancement of MMP-2 activity on breast cancer cells surface

**Figure 4:**
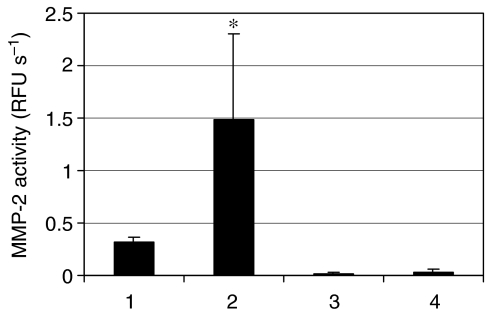
Matrix metalloproteinase (MMP)-2 activity on MDA-MB-231 and MCF-7 cells plated on irradiated Matrigel. Matrigel was irradiated at 0 or 20 Gy and the MDA-MB-231 or MCF-7 cells were plated and incubated for 18 h at 37°C. Then the MEM 0.1% BSA was replaced by the enzymatic buffer A and the MMP-2 activity was measured by adding the fluorogenic peptide MMP Substrate III. (1) MDA-MB-231 cells and 0 Gy; (2) MDA-MB-231 cells and 20 Gy; (3) MCF-7 and 0 Gy and (4) MCF-7 and 20 Gy. ^*^n=3, *P*<0.05.

Irradiated Matrigel increases the expression of MMP-2 as shown by an enhancement of its mRNA and the release of proMMP-2 protein in culture media. We have also determined whether the activity of MMP-2 on the cell membrane of MDA-MB-231 and MCF-7 cells was also increased. The two cell lines were plated on irradiated Matrigel and incubated for 18 h. A fluorogenic peptide cleaved by MMP-2 was then added. Our data demonstrate that irradiation of Matrigel leads to a 4.5-fold increase of MMP-2 activity on the surface of MDA-MB-231 cells, while no MMP-2 activity was measured on the MCF-7 cells (Figure 4).

### Radiation alone did not convert proMMP-2 into active MMP-2

Radiolysis of water by ionising radiation generates the free radicals O_2_^·−^ and ^·^OH. Studies in our laboratory and elsewhere have shown that these free radicals can convert proMMP-2 into active MMP-2 (Saari *et al*, 1992; Paquette *et al*, 2003). Therefore, assays were carried out to determine whether ionising radiation at a dose of 20 Gy could lead to the activation of proMMP-2. The level of MMP-2 activation was determined using a fluorogenic peptide. In parallel, MMP-2 activated by APMA was used as a positive control.

**Figure 5:**
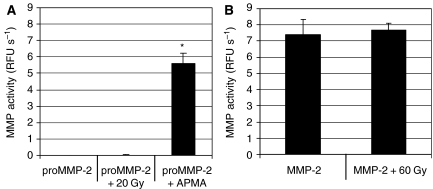
Inability of ionising radiation to directly activate the proMMP-2. (**A**) ProMMP-2 obtained from Calbiochem was irradiated at 20 Gy. As positive control, proMMP-2 was activated by *p-*aminophenylmercuric acetate (APMA). (**B**) Active MMP-2 was irradiated at 60 Gy. The level of matrix metalloproteinase (MMP) activity was determined using the fluorogenic peptide MMP Substrate III. ^*^n=6, *P*<0.05.

As shown in Figure 5A, a dose of 20 Gy did not lead to a detectable activation of proMMP-2. The enzymatic assay was working, as determined after activation of proMMP-2 by the activator APMA. To rule out the possibility that radiation might degrade MMP-2 after the cleavage of the propeptide, active MMP-2 was exposed to 60 Gy. No loss of MMP-2 activity was measured confirming that radiation did not degrade the active MMP-2 (Figure 5B).

## DISCUSSION

After breast-conserving surgery, 39–63% of patients have additional malignant foci scattered throughout the breast (Lagios *et al*, 1981; Holland *et al*, 1985). Radiation therapy after surgery consists typically of irradiation of the whole breast with 50–60 Gy, delivered over 6 weeks. However, the radiation dose is not calculated to eliminate all residual cancer cells. Rather, the radiation dose is calculated to optimise the long-term results with minimal complications (Coles *et al*, 2005). The overall aim of this study was to determine whether ionising radiation could increase the invasion ability of breast cancer cells.

Radiation enhancement of cancer cell invasiveness has already been reported for other cancers. *In vitro* irradiation of human glioma cells increased the expression of MMP-2 and enhanced their invasiveness (Wild-Bode *et al*, 2001; Park *et al*, 2006; Zhai *et al*, 2006). Sublethal *in vitro* irradiation of rat 9L glioma cells resulted in the formation of a greater number of tumour satellites after injection of these irradiated cells into the striatum of rat brain. The ability of radiation to increase the expression of some MMPs and the invasiveness of cancer cells have also been reported for pancreatic cancer cells (Qian *et al*, 2002; Ohuchida *et al*, 2004), melanoma cells (Kaliski *et al*, 2005), rectal carcinoma cells (Speake *et al*, 2005), colon carcinoma cells and osteosarcoma cells (Wang *et al*, 2000).

Cancer cells in the breast are surrounded by the basement membrane, which plays an important role in regulating the expression of numerous genes. In this study, we wanted to determine whether irradiation of Matrigel (a reconstituted basement membrane) could modify the invasive behaviour of breast cancer cells.

Using invasion chambers, we have demonstrated that *in vitro* irradiation of Matrigel at a dose as low as 5 Gy before cells plating enhanced the invasiveness of the highly metastatic MDA-MB-231 breast cancer cells. This radiation enhancement of invasion was associated with the upregulation of pro-invasive gene MMP-2. Indeed, irradiation of Matrigel before plating the cancer cells resulted in an enhancement of proMMP-2 secreted from the two breast cancer cell lines tested in the culture media, as detected using a zymography gel. This enhancement was associated with an increase of MMP-2 mRNA in both cell lines, as quantified by real-time PCR. Supporting the role of MMP-2, radiation enhancement of breast cancer cell invasion was prevented by an MMP-2 inhibitor. These results support the importance of the interactions between breast cancer cells and irradiated Matrigel for the expression of MMP-2.

MT1-MMP and TIMP-2 are required for the conversion of inactive proMMP-2 into active MMP-2. The activation intermediate of MMP-2 matures after binding to α_v_β_3_ integrin which is localised at the cancer cell surface and concentrated at the migrating front (Brooks *et al*, 1996). Irradiated Matrigel increased the expression of both MT1-MMP and TIMP-2, as measured by real-time PCR, and increased MMP-2 activity on the surface of MDA-MB-231 cells. Supporting the role of MT1-MMP, the radiation enhancement of MDA-MB-231 cell invasion was prevented by mixing the cancer cells with an anti-MT1-MMP antibody before plating in the irradiated invasion chamber.

Conversely, no enhancement of invasiveness was observed with the low metastatic MCF-7 cell line. This latter result supports the concept that radiation did not create ‘holes’ in Matrigel, which would facilitate the migration of breast cancer cells. The inability of radiation to enhance the invasion of MCF-7 cells was associated with the absence of MT1-MMP expression, which is required to activate proMMP-2. The MCF-7 cells do not also express the α_v_β_3_ integrin (Deryugina *et al*, 1996) and consequently no MMP-2 activity was detected on surface of this breast cancer cell line. These results suggest that the expression of MT1-MMP is required for the enhancement of breast cancer cell invasion induced by irradiating the Matrigel.

The radiation enhancement of breast cancer cell invasion reported in this study could be related to two pathways. The stimulation of the expression of various MMPs by damaged basement membrane is a normal process observed during wound healing (Gillard *et al*, 2004). Following damage to basement membrane by chemical or physical agents, the production of MMPs by fibroblasts is stimulated resulting in the remoulding and the restoration of basement membrane. The stimulation of MMPs expression is related to the following mechanisms. Basement membrane stores pro-invasive factors such as IGF, EGF and bFGF. These growth factors can be released by agents, which damage the basement membrane. Once released, they can stimulate the expression of MMP-2 and other MMPs (Mira *et al*, 1999; Zhang and Brodt 2003; Lee *et al*, 2004). Our results support the idea that the radiation enhancement of cancer cell invasion was related to the release of pro-invasive factors stored in the Matrigel. Indeed, we have shown that the transfer of conditioned PBS, which was covering the Matrigel during the irradiation, to new non-irradiated invasion chambers resulted in an eightfold increase of MDA-MB-231 cells invasiveness.

An alternative explanation might be that basement membrane is a source of matrikines, which can be unmasked after limited proteolysis or cleavage by free radicals, as generated by ionising radiation. In an adult organism in the resting state, there is little MMP expression and activity (Bosman and Stamenkovic 2003). However, basement membrane fragments or matrikines unmasked by tissue injury can upregulate the expression of MMPs. For example, the *γ*2 subunit DIII fragment of laminin-5 can bind to the cell surface of MDA-MB-231 breast cancer cells and upregulate the expression of MMP-2 and -9 resulting in an increase migratory activity (Schenk *et al*, 2003). The involvement of these two mechanisms in radiation enhancement of cancer cell invasion deserves to be tested in subsequent studies.

Radiotherapy is an efficient modality to treat malignant breast tumours and other types of solid tumours. However, the present study and previous reports published by other teams demonstrate that radiation can also increase the invasion capacity of cancer cells. To improve the efficacy of radiotherapy, this phenomenon must be further studied to elaborate therapeutic modalities to prevent radiation enhancement of cancer cell invasion.

## Figures and Tables

**Table 1:** Effects of irradiated Matrigel on MMP-2, MT1-MMP and TIMP-2 expression

	**Enhancement ratio (20 Gy per 0 Gy^a^)**	
	**MDA-MB-231**	**MCF-7**
MMP-2	3.90 (±0.28)^*^	1.58 (±0.16)^*^
MT1-MMP	3.07 (±0.11)^*^	ND
TIMP-2	1.59 (±0.30)^*^	1.40 (±0.30)

a The levels of mRNA were normalized according to the internal standard GAPDH. The enhancement ratio was calculated by dividing the normalized level measured at 20 Gy by the level at 0 Gy. Data represent the average of 4–6 assays±standard deviation.

MMP-2=matrix metalloproteinase-2; MT1-MMP=membrane type 1 matrix metalloproteinase; TIMP-2=tissue inhibitor of metalloproteinase-2; ND=not detected.

^*^n=4-6. *P*<0.05.

**Table 2:** Effect of Matrigel irradiation on the invasiveness of MDA-MB-231 cells

**No.**	**Dose (Gy)**	**Conditions**	**Average number of cells migrating through Matrigel (s.d.)^a^**
1	0	No irradiation	6.3 (±2.3)
2	20	Invasion chamber irradiated without PBS	24.0 (±9.6)^*^
3	20	Invasion chamber irradiated with PBS	54.0 (±13.0)^*^
4	0	PBS was removed after irradiation of Matrigel and then added to new non-irradiated chambers before cells plating	57.0 (± 28.6)^*^

PBS=phosphate-buffered saline; s.d.=standard deviation.

Invasion chambers were treated as indicated. MDA-MB-231 cells (1 × 10^4^) were added after irradiation of the invasion chambers and incubated for 6 h in MEM 0.1% BSA. The same volume of PBS was added to the other invasion chambers (condition nos. 1, 2 and 4) before cells plating.

^*^n=3. Student's-*t* test: condition no. 1 *vs* 2, *P*=0.003; condition no. 1 *vs* 3, *P*=0.026; conditions no. 1 *vs* 4, *P*=0.023.

a Average number of invasive cells observed per microscope field.
